# Crystal Structures of HIV-1 gp120 Envelope Glycoprotein in Complex with NBD Analogues That Target the CD4-Binding Site

**DOI:** 10.1371/journal.pone.0085940

**Published:** 2014-01-28

**Authors:** Young Do Kwon, Judith M. LaLonde, Yongping Yang, Mark A. Elban, Akihiro Sugawara, Joel R. Courter, David M. Jones, Amos B. Smith, Asim K. Debnath, Peter D. Kwong

**Affiliations:** 1 Vaccine Research Center, National Institute of Allergy and Infectious Diseases, National Institutes of Health, Bethesda, Maryland, United States of America; 2 Department of Chemistry, Bryn Mawr College, Bryn Mawr, Pennsylvania, United States of America; 3 Department of Chemistry, University of Pennsylvania, Philadelphia, Pennsylvania, United States of America; 4 Laboratory of Molecular Modeling and Drug Design, Lindsey F. Kimball Research Institute of the New York Blood Center, New York, New York, United States of America; NCI-Frederick, United States of America

## Abstract

Efforts to develop therapeutic agents that inhibit HIV-1 entry have led to the identification of several small molecule leads. One of the most promising is the NBD series, which binds within a conserved gp120 cavity and possesses *para*-halogen substituted aromatic rings, a central oxalamide linker, and a tetramethylpiperidine moiety. In this study, we characterized structurally the interactions of four NBD analogues containing *meta*-fluoro substitution on the aromatic ring and various heterocyclic ring replacements of the tetramethylpiperidine group. The addition of a *meta*-fluorine to the aromatic ring improved surface complementarity and did not alter the position of the analogue relative to gp120. By contrast, heterocyclic ring replacements of the tetramethylpiperidine moiety exhibited diverse positioning and interactions with the vestibule of the gp120 cavity. Overall, the biological profile of NBD-congeners was modulated by ligand interactions with the gp120-cavity vestibule. Herein, six co-crystal structures of NBD-analogues with gp120 provide a structural framework for continued small molecule-entry inhibitor optimization.

## Introduction

The HIV-1 viral spike is composed of three copies of the gp120 envelope glycoprotein attached non-covalently to three copies of the gp41 transmembrane molecule [1], [2], [3]. Binding of the primary cell surface receptor, CD4, to the gp120 component of the viral spike exposes and/or induces the formation of a site for co-receptor binding (either CCR5 or CXCR4) [4], [5], [6], [7]. Co-receptor binding to gp120 triggers additional conformational changes in the trimer spike, which leads ultimately to a fusion of the viral and host cell membranes [8], [9], [10]. Several HIV-1 entry inhibitors that block the progression of this multi-step fusion processes have been developed. Enfuvirtide, a biomimetic peptide with a molecular weight of ∼4.5 kDa, was the first FDA-approved fusion inhibitor that binds to gp41 and blocks the gp41 fusion process [11]. The small molecule, maraviroc (MW = 513.7 Da) [12], [13], represents another class of FDA approved HIV-1 entry inhibitor, which binds CCR5 and in turn induces a conformation that is not recognized by gp120 [14], [15], [16], [17]. However, small molecules that directly target the conserved CD4-bi ding site on gp120 and thus block HIV-1 cell entry have not been fully developed.

**Figure 1:**
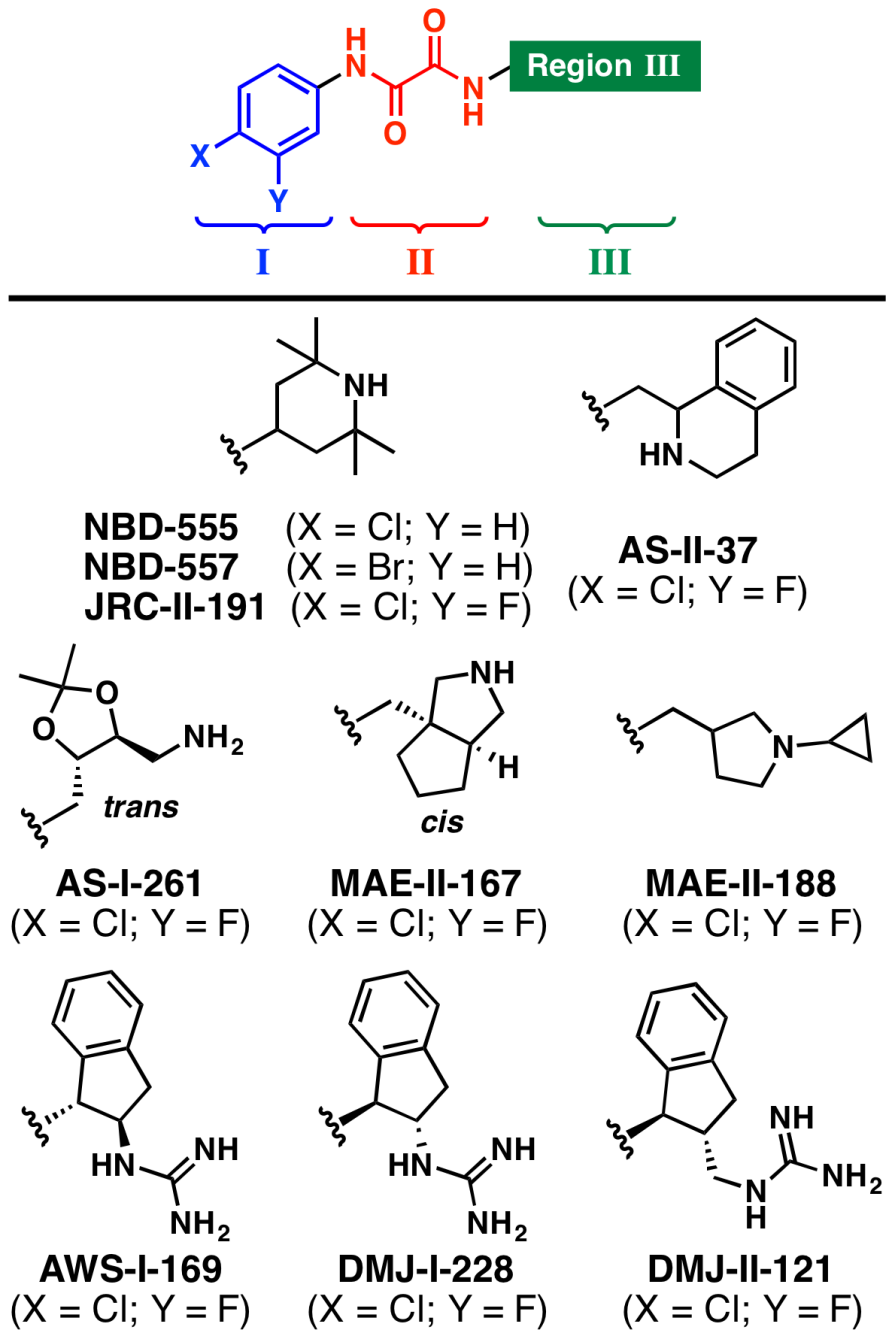
The chemical structures of NBD analogues that target the CD4 binding site of gp120. The general structure of the NBD congeners is shown, defining Regions I (blue), II (red), and III (green). The diverse Region III scaffolds crystalographically characterized in complex with gp120 are shown below.

The HIV-1 attachment inhibitors, NBD-556 and NBD-557, were identified by screening a drug-like small molecule chemical library [18]. The NBD chemotype consists of three essential regions: Region I, a *para*-substituted aromatic ring; Region II, an oxalamide linker; and Region III, a tetramethylpiperidine heterocyclic ring system (Figure 1). In cell-based assays, these small molecules inhibit HIV-1 entry with IC_50_ values of 58.5 to >100 µM and bind gp120 with 3–5 µM affinity (Table S1) [18]. Isothermal titration calorimetry studies reveal that NBD-556 and NBD-557 bind gp120 with a large unfavorable entropy change, comparable to that observed for CD4-gp120 interaction, suggesting that NBD-556 and NBD-557 induce full-length monomeric gp120 to the CD4-bound conformation [19]. Both soluble CD4 (sCD4) and NBD-556 inactivate HIV-1 by prematurely triggering active but transient intermediate states of the envelope glycoprotein that rapidly and irreversibly decay to conformations that are no longer fusion-active [20]. Therefore, sCD4 and small molecules that mimic the host cell CD4 receptor may inhibit HIV-1 infection either by preventing attachment to CD4 on the cell surface or by inducing a short-lived activated state [20]. Modeling [21] and mutagenesis studies [22] suggested NBD-556 and NBD-557 bind at an unusual interfacial juncture of gp120 called the “Phe 43 cavity.” This was confirmed by the co-crystal structure of a clade C_1086_ gp120 core in complex with NBD-556 [23]. The NBD-556:gp120 crystal structure provided a framework for design and synthesis of a subsequent generation of NBD analogues [24], [25]. Several recently disclosed Region III congeners exhibit both increased affinity for gp120 and improved potency against selected clade B and C viruses relative to NBD-556 [24], [25], [26]. An exploration of structure-activity relationships [27] demonstrated that variation of the Region III moiety not only influences compound binding affinity, but also modulates the capacity of NBD-derived compounds to enhance viral infectivity in cells lacking the CD4 receptor (CD4^−^CCR5^+^ Cf2Th cells) [21], an undesired trait for the development of viral entry inhibitors. Indeed, Region III analogues possessing a *trans*-1,2-indane and guanidinium functionality [24], [26] do not enhance CD4 independent viral entry in contrast to NBD-556, NBD-557 and other Region III analogues. Instead, these Region III congeners are full antagonists of the viral entry process [26], [28]. Herein, we present the high-resolution structures of NBD-557 in complex with clades B_YU2_ and in complex with clade A/E_93TH057_ gp120 core, as well the structures of four Region III NBD-congeners, AS-II-37, AS-I-261, MAE-II-167, and MAE-II-188 (Figure 1), in complex with the clade A/E_93TH057_ gp120 core_e_. We further describe how the diverse binding modes revealed in the six small-molecule:gp120 complexes provide a framework by which to optimize entry inhibitors possessing the NBD-chemotype as anti-HIV-1 therapeutics.

## Results

### Crystallization of NBD-557 in complex with gp120 core

**Figure 2:**
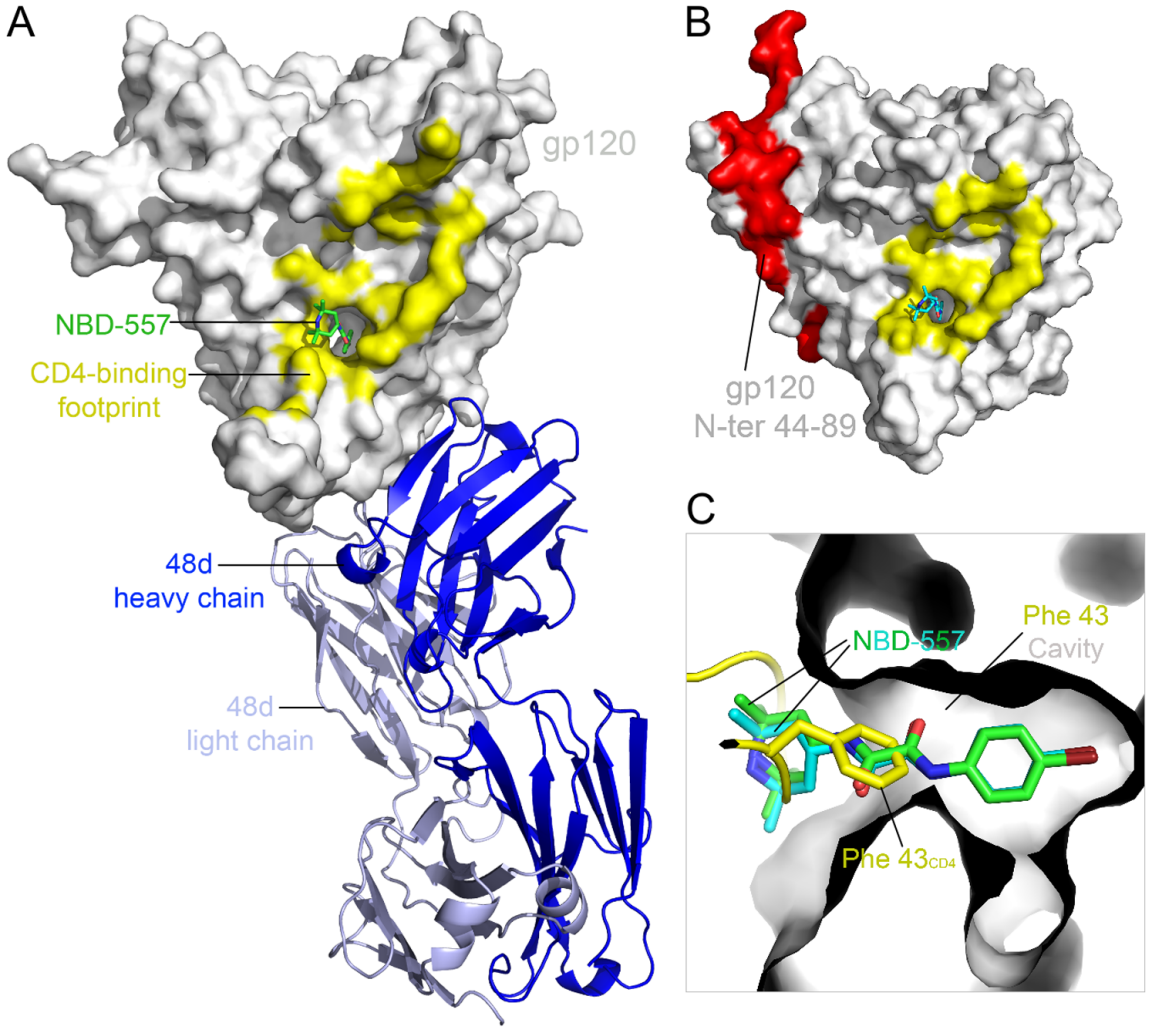
Structures of HIV-1 gp120 core in complex with NBD-557. (A) YU2 gp120 coreV3s (surface representation in grey) in complex with NBD-557 (stick representation in green) and Fab 48d depicted in a ribbon diagram (light chain in light blue and heavy chain in blue). (B) NBD-557 (stick representation in cyan) binds the Phe 43_CD4_ cavity on clade A/E_93TH057_ gp120 core_e_. Area colored red represents N-terminal residues (44–89), which are missing in gp120 core in (A). CD4 footprints on gp120 are colored in yellow in (A) and (B). (C) Superposition of NBD-557- and Fab 48d- bound YU2 gp120, NBD-557-bound clade A/E_93TH057_ gp120 core, and the CD4-bound gp120. Two NBD-557 (green and cyan) and the side chain of Phe 43_CD4_ (yellow) in the cavity are highlighted.

To crystallize NBD-557 in complex with gp120 core, we initially employed the same approach used for YU2 gp120:sCD4:CD4i antibody complex crystallization [29], except for using NBD-557 in place of sCD4. Unlike sCD4, NBD-557 binding did not yield stable gp120 core:NBD-557:CD4i antibody ternary complexes (Figure S1). Therefore, we devised a more stable gp120 core as reported previously [23]. The new core, coreV3s (“s” stands for substitution), formed a stable ternary complex with NBD-557 and 48d Fab (Figure S1). The complex was crystallized using the hanging-drop vapor diffusion method by mixing equal amount of the complex and the reservoir solution containing 16–20% PEG 3350. The structure was solved at 2.5 Å resolution with molecular replacement (Figure 2 and Table 1). Concurrent with this study, our endeavor to crystallize unliganded HIV-1 gp120 core led us to identify clade A/E_93TH057_ extended core (core_e_), which produced well diffracting crystals in the absence of sCD4 or CD4i antibodies [23]. The clade A/E_93TH057_ gp120 core_e_, however, required mutation of His 375 to Ser (H375S) to co-crystalize with small molecules as reported previously [24]. We used this gp120 core_e_ H375S variant and co-crystallized with NBD-557 or racemic mixtures of (±)-AS-II-37, (±)-AS-I-261, (±)-MAE-II-167, or (±)-MAE-II-188. Here, we report six gp120 structures in complexes with these small molecules determined at 1.9–2.4 Å resolution (Table 1).

**Table 1:** Data collection and refinement statistics.

	YU2 core_e_:	93TH057 core_e_:	93TH057core_e_:	93TH057 core_e_:	93TH057 core_e_:	93TH057 core_e_:
	48d:NBD-557	NBD-557	AS-II-37	AS-I-261	MAE-II-167	MAE-II-188
**Data collection**						
Space group	P2_1_2_1_2_1_	P2_1_	P2_1_	P2_1_	P2_1_	P2_1_
Cell dimensions						
*a*, *b*, *c* (Å)	53.4,109.7,130.6	64.6,68.2,94.0	64.8,68.4,93.9	64.7,68.0,94.3	64.5,68.5,94.1	64.6,68.2,93.9
α, β, γ (°)	90,90,90	90.0,91.4,90.0	90,91.9,90.0	90,91.4,90	90,91.2,90	90,91.4,90
Resolution (Å)	49.4-2.5(2.54-2.5)	50-2.1(2.14-2.1)	50-2.5(2.54-2.5)	50-1.94(1.97-1.94)	50-2.2(2.24-2.2)	50-2.4(2.44-2.4)
*R*_sym_	0.155 (0.497)	0.076 (0.447)	0.085 (0.432)	0.062 (0.393)	0.062 (0.402)	0.076 (0.401)
*I*/σ*I*	11.04 (2.2)	14.8 (1.49)	17.6 (1.46)	22.0 (2.23)	19.6 (2.13)	16.3 (1.7)
Completeness (%)	89.7 (62.9)	88.4 (46.8)	93.9 (60.0)	95.4 (63.4)	97.1 (80.3)	83.6 (27.9)
Redundancy	4.6 (1.8)	3.3 (2.3)	3.0 (1.8)	4.4 (2.6)	3.5 (2.6)	3.5 (2.2)
**Refinement**						
Resolution (Å)	49.4-2.5	25.0-2.1	28.1-2.5	26.3-1.94	27.7-2.2	28.4-2.4
Unique reflections	27,496	43,496	31,053	58,216	40,942	26,934
*R*_work_/*R*_free_	24.3/28.8	20.5/24.6	20.8/26.0	18.1/21.5	19.9/23.8	20.8/27.1
No. atoms						
Protein	5,533	5,308	5,308	5,308	5,308	5,308
Ligand/ion	135	384	388	386	397	382
Water	103	231	84	378	207	67
*B*-factors (Å^2^)						
Protein	72.5	54.9	63.1	53.5	55.9	62.7
Ligand/ion	90.9	78.3	94.3	72.5	81.9	91.4
Water	50.7	46.2	46.0	50.0	46.3	44.8
R.m.s. deviations						
Bond lengths (Å)	0.002	0.002	0.003	0.003	0.003	0.002
Bond angles (°)	0.490	0.650	0.700	0.803	0.718	0.661
PDB ID	4DVR	4DVS	4DVT	4DVV	4DVW	4DVX

*Values in parentheses are for highest-resolution shell.

### Structures of NBD-557 in complex with gp120 core-48d or gp120 core_e_

**Figure 3:**
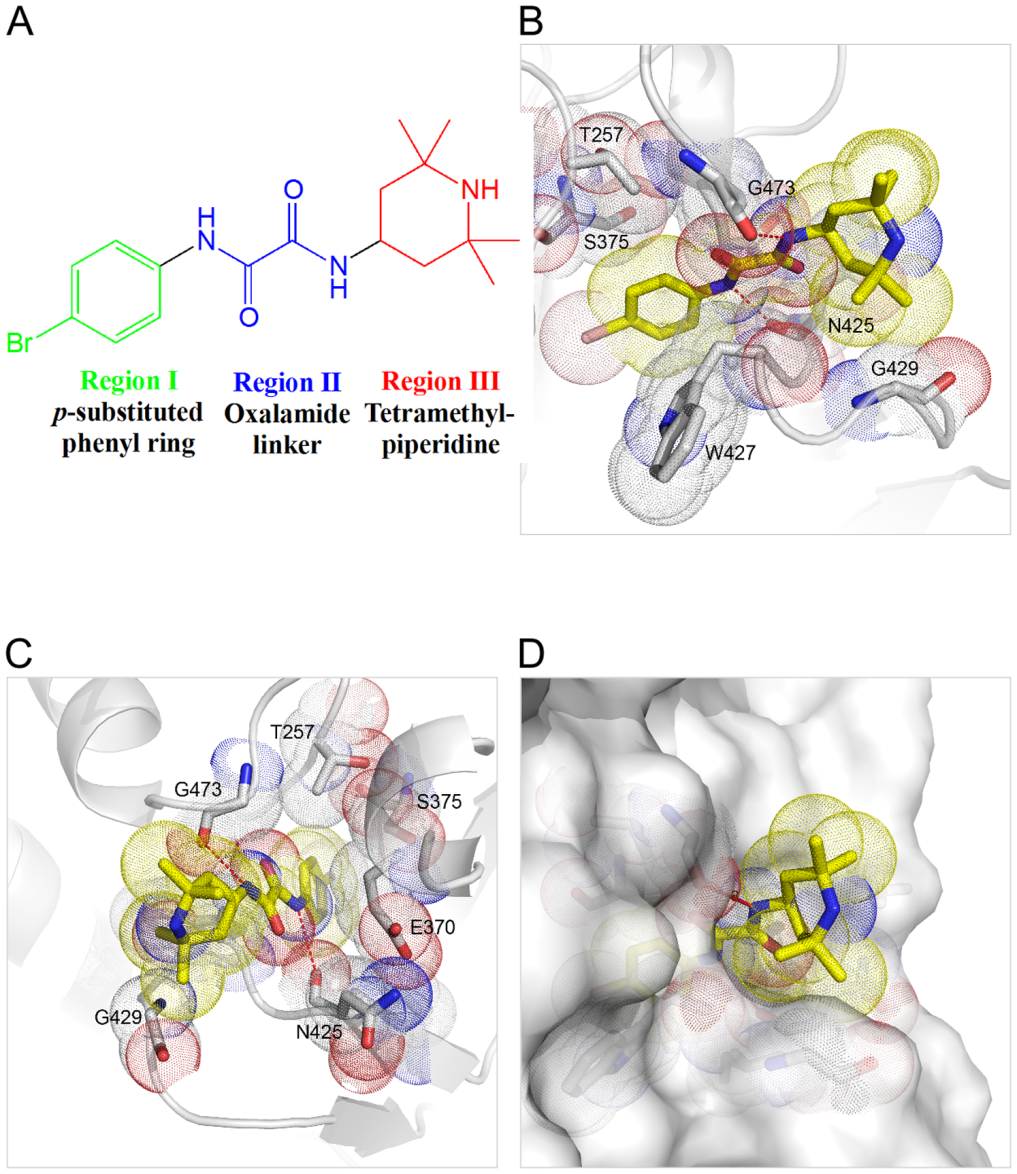
Detailed interactions of NBD-557 with gp120. (A) The chemical structure of NBD-557, defining Regions I, II, and III. (B, C) NBD-557 interactions with clade A/E_93TH057_ gp120 in the cavity. (D) The tetramethylpiperidine ring resides in the cavity vestibule and does not make optimal contacts with gp120 surface.

In both the NBD-557:clade B_YU2_ gp120 coreV3s:48d (Figure 2A) and the NBD-557: A/E_93TH057_ gp120 core_e_ (Figure 2B) complexes, the Region I *para*-bromo substituted aromatic ring was inserted most deeply within the Phe 43 cavity (Figure 2C). Comparison of the NBD-557:clade B_YU2_ gp120 core V3s:48d complex with the NBD-557:clade A/E_93TH057_ gp120 core_e_ complex demonstrated that the conformation of NBD-557 was nearly identical, with only a slight deviation of the tetramethylpiperidine *gem*-dimethyl groups (Figure 2C). Superposition of the NBD-557:gp120 and CD4:gp120 complexes revealed that the position of the tetramethylpiperidine ring and oxalamide linker of NBD-557 overlapped with the backbone and the Phe 43_CD4_ side chain, revealing that the *para*-substituted phenyl ring of NBD-557 binds more deeply into the cavity compared to the Phe 43_CD4_ side chain (Figure 2C). In the NBD-557:A/E_93TH057_ gp120 core_e_ complex, residues Thr 257, Glu 370, Ser 375, and Ile 424 made contacts with the Region I *para*-brominated aromatic ring of NBD-557 (Figure 3), while Trp 427 made an aromatic-aromatic π-stacking interaction with Region I (Figure 3B). Along the neck of the Phe 43 cavity, the nitrogen atoms of the Region II oxalamide linker formed hydrogen bonds with backbone carbonyls of Asn 425 and Gly 473 (Figure 3A–C). In Region III, the *gem*-dimethyl group at position 6 of the tetramethylpiperidine ring formed hydrophobic contacts with the Gly 429 backbone Cα atom on gp120 (Figure 3B and 3C). The tetramethylpiperidine ring made additional hydrophobic contacts with the gp120 surface in the cavity vestibule (Figure 3D). Similar interactions were observed in the NBD-557:clade B_YU2_ gp120 coreV3s:48d complex, with two additional contacts formed with Asp 474 and Met 475. Overall, the structures indicate that only the oxalamide linker of NBD-557 makes direct hydrogen bonds with gp120 (Figure 3B and 3C). Moreover, well-defined interactions were not observed between gp120 and either the Region I, *para*-brominated aromatic ring, or the Region III tetramethylpiperidine ring (Figure 3D). These observations suggested that further improvement in surface complementarity by incorporation of specific protein-ligand interactions within the substituted aromatic ring of Region I, and/or exploration of suitable replacements for the tetramethylpiperidine of Region III should increase the binding affinity of NBD congeners.

### Comparison of ligand interactions within the Phe 43 cavity in gp120

To understand the structural basis for diverse effects of Region I aromatic ring modifications on gp120 binding and neutralization potency, we compared gp120 structures in complex with NBD-557 and bound to NBD analogues AS-II-37, AS-I-261, MAE-II-167, or MAE-II-188 (the enantiomer that preferentially bound gp120 was determined from the structures, as described below). The parental NBD compounds possessed a *para*-halogen substituent on the aromatic ring of Region I, whereas the latter four NBD analogues possessed a *para*-chloro, *meta*-fluoro substituted aromatic ring. Madani et al. previously reported that modifications of the Region I aromatic ring greatly influence NBD-analogue affinity for gp120 [21].

**Figure 4:**
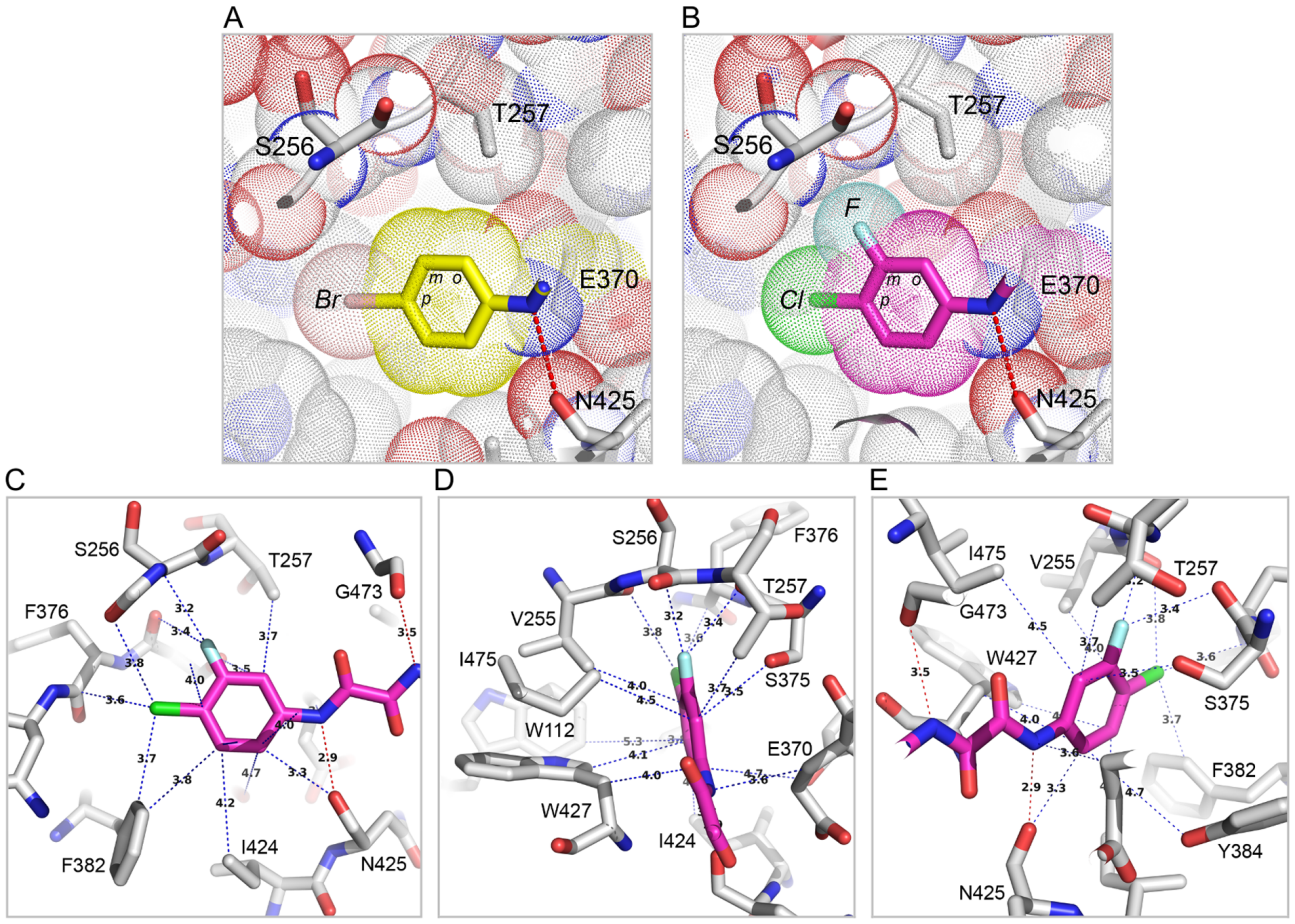
Comparison of Region I and II of NBD analogues in the Phe 43 cavity. (A) The *para*-bromo substituted phenyl ring of NBD-557. (B) The *para*-chloro and *meta*-fluoro substituted phenyl ring of AS-I-261. The fluorine substitution at the *meta* position of AS-I-261 fills the space formed near Ser 256 and Thr 257 in NBD-557 bound Phe 43 cavity (A). *Ortho* substitution of the aromatic ring would not be tolerated in either complex, because of steric clash with gp120. (C–E) Residues on gp120 that make contacts with the Region I and II of AS-I-261. Hydrogen bonds are noted in red dotted lines, and atoms that are within the van der Waals radius (>5 Å) are noted in blue dotted lines.

The structures of these four analogues (AS-II-37, AS-I-261, MAE-II-167, and MAE-II-188) in complex with clade A/E_93TH057_ gp120 core_e_ (H375S) revealed that the Region I and II moieties within the Phe 43 cavity exhibit nearly identical poses to NBD-557. In Region I, the *meta*-fluoro substitution on the aromatic ring of AS-II-37, AS-I-261, MAE-II-167, and MAE-II-188 visually increased shape complementarity between Region I by filling the gp120 Phe 43 cavity near residues Val 255 and Ser 375. This same area in the NBD-557 bound-gp120 cavity was unoccupied (Figure 4A and 4B). The improved surface-complementarity may provide a structural explanation for the previous finding that the *para*-chloro- and *meta*-fluoro-substituted aromatic ring enhances JRC-II-191 binding affinity for YU2 gp120 5-fold and JRC-II-191 inhibits HIV-1_YU2_ infection of cells expressing CD4 and CCR5 more potently than NBD-556 does [21], [27]. A Cf2Th-CD4-CCR5 cell viability assay showed that use of 100 µM of DMJ-I-228, an NBD analogue which also possessed a *para*-chloro- and *meta*-fluoro-substituted aromatic ring, does not alter the cell growth dynamics, suggesting that chloro- or fluoro- substitution does not exert cytotoxicity [24]. Together, these suggest that the improved surface-complementarity between the *meta*-fluoro-substituted aromatic ring and the Phe 43 cavity correlates with the improved pharmacokinetics of these NBD analogues.

Among the four analogue complexes, the region I *para*-chloro and *meta*-fluoro substituted aromatic ring bound with nearly identical positions. As AS-I-261-bound gp120 structure was determined at the highest resolution (1.9 Å), the AS-I-261 complex was used to describe interactions within the Phe 43 cavity (Figure 4). As observed in the NBD-557:gp120 structure, Trp 427 made aromatic-aromatic π-stacking interactions with the *para*-chloro and *meta*-fluoro substituted aromatic ring of AS-I-261 (Figure 4D and 4E). Orthogonal dipolar interactions between the *meta*-fluoro atom and carbonyls of Val 255 and Ser 375 were also observed (Figure 4D). However, close packing of gp120 side chains adjacent to the Region I aromatic ring suggested that substitution at the *ortho*-position would create steric clashes with Thr 257 (Figure 4B–D), and provided a structural basis for ablated binding of the *ortho*-substituted NBD analogues to gp120 [21], [27]. In Region II, the two nitrogen atoms on the oxalamide linker made hydrogen bonds with the backbone carbonyls of Asn 425 and Gly 473, as found in the NBD-557: A/E_93TH057_ gp120c core_e_ structure (Figure 4C–E). Thus, the binding modes of Region I aromatic rings within the Phe 43 cavity revealed in both this study and studies of CD4-mimetic miniproteins [30], [31], [32], [33] suggest that NBD analogues capable of increasing shape complementarity within the Phe 43 cavity should be explored further.

### Diverse interaction modes between Region III of NBD analogues and gp120

**Figure 5:**
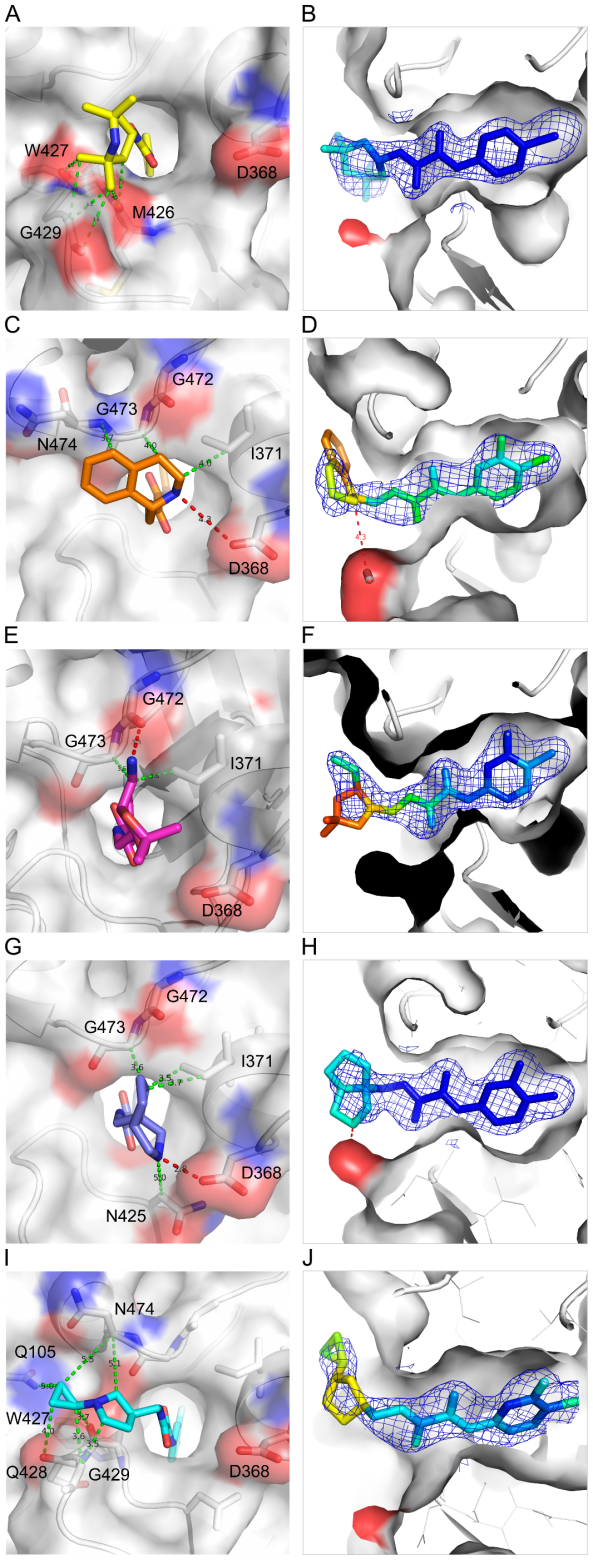
Diverse interaction modes of NBD-analogue Region III with gp120. Close-up views of NBD-557 (A, B), AS-II-37 (C, D), AS-I-261 (E, F), MAE-II-167 (G, H), and MAE-II-188 (I, J) in the Phe 43 cavity. Region I and II reside inside the Phe 43 cavity. The Region III of the analogues protrudes outside the cavity in diverse conformations. All atoms on gp120 within 5 Å distance to the Region III of the analogues are shown with dotted lines; hydrogen bonds in red and two atoms within the van der Waals radius in green dotted lines. (B, D, F, H, and J) NBD analogues in *2fo-fc* electron density map colored by the *B*-factor. The color scale ranges from blue to red for *B*-factors of >40 to <150 Å^2^.

**Figure 6:**
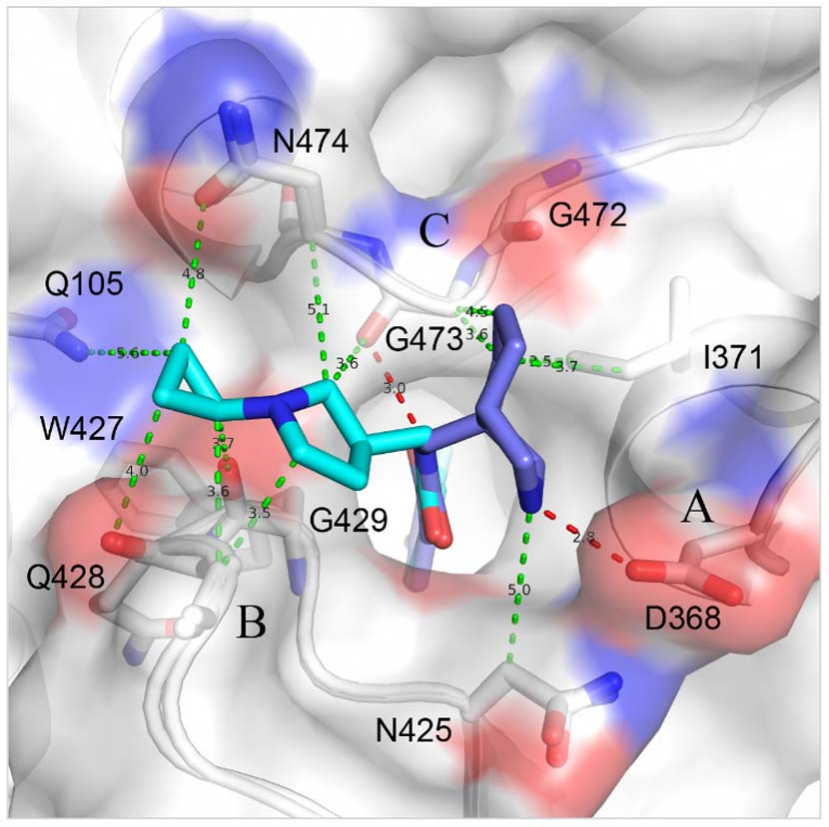
Design strategy. Superposition of MAE-II-167 (blue) and MAE-II-188 (cyan) at the Phe 43 cavity suggests a model compound that forms a hydrogen bond with Asp 368 in Area A. The model makes hydrophobic contacts with the tip of β20/21 in Area B. A successful design strategy for a potent drug should include additions of an amine group that will interact with Asp 368 by a salt bridge, a longer hydrophobic tail that will satisfy hydrophobic interactions in Area B, and a moiety that expands contacts in Area C.

Four NBD analogues, synthesized as racemic mixtures, (±)-AS-II-37, (±)-AS-I-261, (±)-MAE-II-167, and (±)-MAE-II-188 (Figure 1), were selected for structural studies as each possessed a Region III amine for potential interaction with essential gp120 residue, Asp 368 and to relate structure and function of the diverse chemotypes and the capacity to either inhibit HIV-1 entry or enhance CD4-independent viral infectivity [27]. As expected, the structures of these four compounds in complex with gp120 revealed a variety of interaction modes to exist between the analogues' Region III moiety and the gp120 Phe 43 cavity vestibule (Figure 5). Electron densities of the Region III moieties were, however, not as well defined as those of Regions I and II. Atoms in Region III displayed high *B*-factors (Figure 5B, D, F, H, and J), indicating this region to be flexible. This may result from conformational flexibility of the Region III moiety, the lack of interactions between the Region III moiety and gp120, and/or from binding of individual enantiomers present in the racemic mixtures employed for crystallization. Even so, fitting of each enantiomer in the electron densities clearly indicated the preferred binding of the (*S,S*)-AS-I-261, (*S,S*)-MAE-II-167, and (*R*)-MAE-II-188 enantiomers (Figure S3). The poor electron density of the Region III tetrahydroisoquinoline of AS-II-37 (Figure 5D) led us to model AS-II-37 in three conformations: one for the (*R*)-enantiomer and two for the (*S*)-enantiomer (Figure S2 and S3). We selected a conformation of the (*S*)-enantiomer in which the tetrahydroisoquinoline nitrogen was positioned to make a weak electrostatic interaction (4.3 Å) with Asp 368 of gp120 (Figure S2). Of note, only one other analogue, (*S*,*S*)-MAE-II-167 interacted with Asp 368, with the nitrogen atom from the pyrrole ring forming a hydrogen bond with Asp 368. In contrast the cyclopropyl-substituted pyrrolidine of (*R*)-MAE-II-188 and the ethyl-amine nitrogen atom of (*S*,*S*)-AS-I-261 bound to the opposite face of the Phe 43 cavity vestibule. In the case of AS-I-261, a hydrogen bond is formed with outer domain residue Gly 472. Importantly, none of the four analogues [(*S*)-AS-II-37, (*S*,*S*)-AS-I-261, (*S*,*S*)-MAE-II-167, and (*R*)-MAE-II-188] exhibited binding to Asp 368 via a water-mediated network, as reported for the functional antagonists AWS-I-169 and DMJ-I-228 or a hydrogen bond to the backbone carbonyl of Met 426 as in DMJ-II-121 (Figure 1) [24], [26]. Overall, the Region III binding modes for the (*S*)-AS-II-37, (*S*,*S*)-AS-I-261, (*S*,*S*)-MAE-II-167, and (*R*)-MAE-II-188 in complex with gp120 reveal three major areas within the cavity vestibule that can be exploited to maximize gp120-small molecule interactions. These include: i) Asp 368 on the CD4-binding loop (Figure 6, Area A); ii) the tip area of β20/21 strand (Figure 6, Area B), and iii) the outer-to-inner domain exit loop (Figure 6, Area C). Compounds possessing either a basic amine or a guanidinium in Region III are desired, as they can interact with Asp 368 via a hydrogen bond or electrostatic interaction in Area A. This concept has already been proven to increase binding affinity of (*S,S*)-DMJ-I-228 and (*R,R*)-DMJ-II-121 whose guanidinium groups in the Region III interact with Asp 368 or Met 426, respectively [24], [26] and a 5-(hydroxymethyl)-4-methylthiazol-2-yl)(piperidin-2-yl)methyl) region III containing analogue [25].

### Relationships between Region III binding modes and activation of viral infectivity

**Figure 7:**
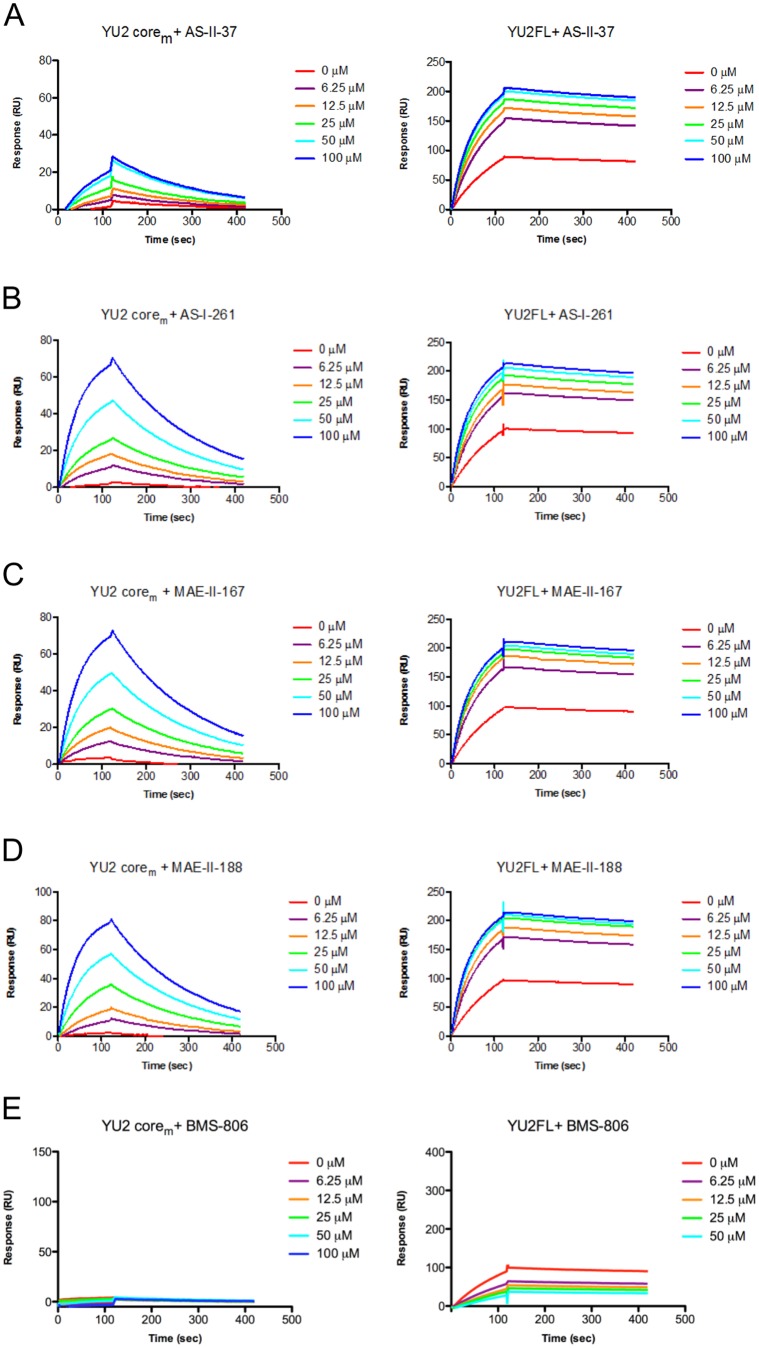
NBD analogue binding to gp120 enhances 17b-gp120 interaction. (A–D) 100 nM of gp120 core_min_ or full-length gp120 in the presence of 0–100 µM of NBD analogues was passed over 17b antibody immobilized on a CM5 chip. The binding of gp120 to 17b increased, in a concentration dependent manner, in response to treatment with the NBD analogues. (E) In contrast, the presence of BMS-806, a small molecule inhibitor that has known not to induce gp120 conformation, did not enhance gp120-17b interaction.

A major undesired trait of NBD analogues as HIV-1 entry inhibitors is their capacity to activate viral infectivity in CD4-negative cells. To understand the underlying mechanisms of activation of HIV-1 infectivity in CD4-negative cells in the presence of select NBD analogues, we asked if the capacity of the four analogues to induce gp120 conformational change to the CD4-bound state might account for observed differences in viral infectivity. To address this question, we employed surface-plasmon resonance (SPR) and measured the capacity of NBD-557, (±)-AS-II-37, (±)-AS-I-261, (±)-MAE-II-167, and (±)-MAE-II-188 to increase the binding affinity of either the minimal core (core_min_) or full-length gp120 for the 17b antibody, which recognizes gp120 in the CD4-bound conformation. We chose gp120 core_min_ over core_e_ for SPR measurement because the core_e_ is already in the CD4 bound conformation [23], and the effect of these small molecules on 17b-gp120 interaction is undetectable. Congeners (±)-AS-II-37, (±)-AS-I-261, (±)-MAE-II-167, and (±)-MAE-II-188, which displaying similar affinities for full-length gp120, equally enhanced monomeric core_min_ and full-length gp120 binding to 17b (Figure 7A–E). These enhancements of 17b binding were comparable to those observed with NBD-557 and DMJ-I-228 [24], except for with (±)-AS-II-37, which exerted somewhat reduced enhancement for core_min_ binding to 17b (Figure 7A). Overall, the SPR experiment suggested that i) the conformation of the core_min_ gp120 is quite different from that of the core_e_ and ii) these small molecules are indeed capable of enhancing gp120 binding to 17b. However, the SPR measurements failed to provide a clear explanation for how the small molecule Region III moieties exert diverse effects on viral infectivity in CD4 negative cells. For example, AS-I-261 and DMJ-I-228 equally enhanced monomeric gp120 binding to 17b, while these two compounds showed very different viral infectivity; AS-I-261 activated viral infectivity in the CD4-neagative cells, but DMJ-I-228 did not (Table S1). Thus, the lack of correlation among Region III binding modes, SPR study, and viral infectivity led us to speculate that the undesired enhancement of CD4-independent HIV-1 infection may instead be modulated by interactions between Region III of the small molecule and gp120 that alter the conformations of variable loop elements in the viral spike, which were not revealed in the structures in the current study.

### HIV-1 strains intrinsically resistant to the NBD-like compounds

**Figure 8:**
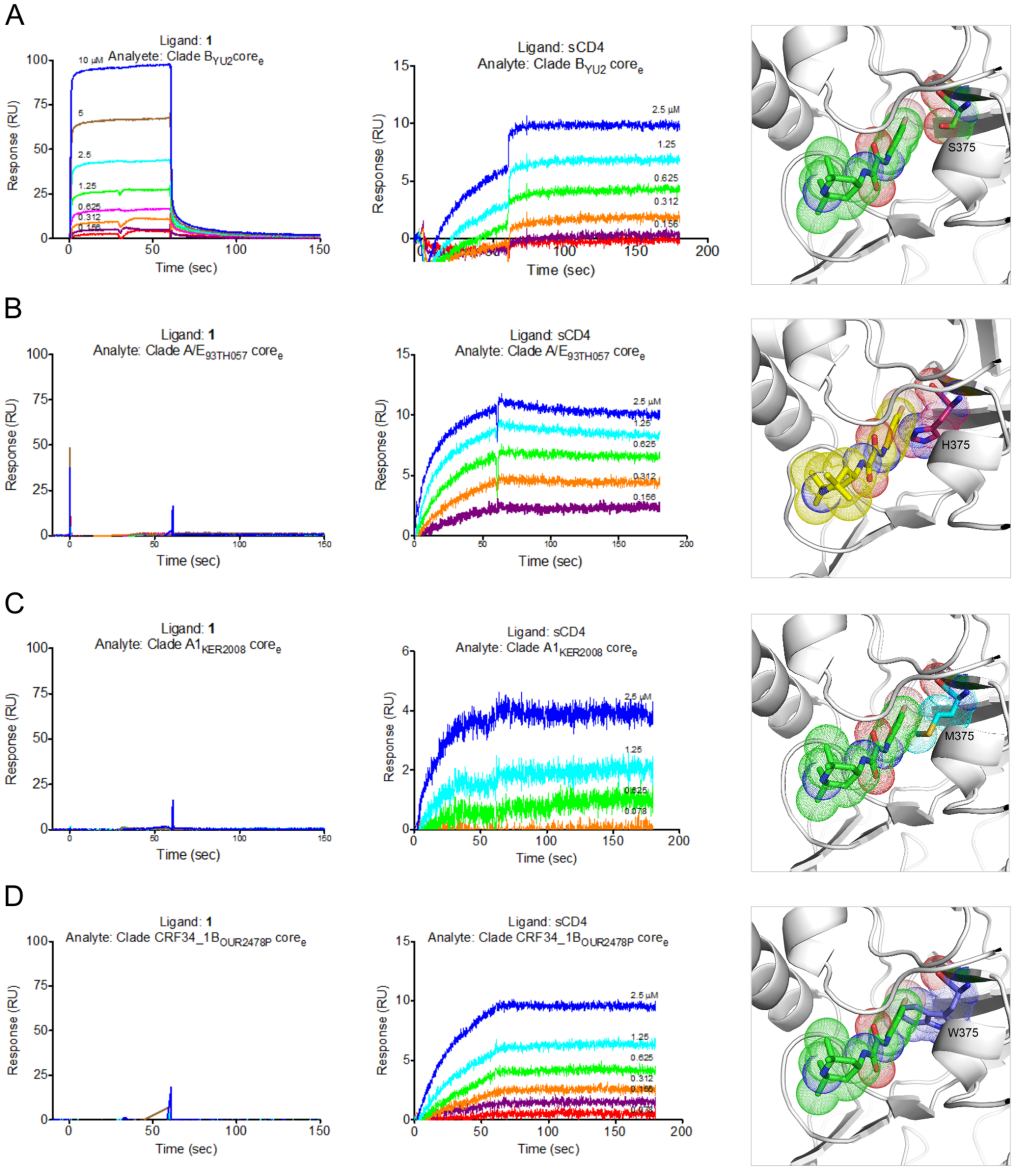
NBD analogues are unable to bind Ser 375 variants of gp120s. (A) Clade B_YU2_ gp120 core_e_ binds both NBD-556(Ligand:**1**) and sCD4. Ser 375 was shown in the NBD-557 bound cavity. (B) Clade A/E_93TH057_ gp120 core_e_ does not show detectable binding to NBD-556 immobilized onto a CM5, but binds to sCD4. NBD-557 modeled into the Phe 43 cavity of clade A/E gp120 clashes with His 375. (C) Clade A_KER2008_ gp120 core_e_ does not bind NBD-556, but does bind sCD4. Met 375 modeled into NBD-557-bound YU2 gp120 clashes with NBD-557. (D) Clade CRF34_1_OUR2478P_ gp120 core_e_ does not bind NBD-556, but binds sCD4. NBD-557 clashes with Trp 375 in the Phe 43 cavity.

Our structures reveal that Region I and II of the NBD congeners fit deeply within the Phe 43 cavity. As such, mutations that alter the shape of the cavity will also affect binding of the small molecules to gp120. One residue in close proximity to Region I and II of the NBD congeners is Ser 375 (*vide supra*). For example, as previously described full-length gp120 variants possessing the Ser 375 to Trp mutation (S375W) inefficiently bind NBD-556 [21]. In addition, NBD-557 does not enhance CD4-independent viral entry when the virus expressing the S375W Env gp120 is incubated with the target cells [21]. In several group O, N, or M HIV-1 viruses, Ser 375 of gp120 is replaced with either Met or His. To test whether NBD-556 binds these gp120 variants, we immobilized NBD-556 and sCD4 on a Biacore sensor chip and passed the gp120 variants over these ligands [23]. As expected, these gp120 variants did not show any detectable binding to NBD-556, although each variant did bind sCD4 (Figure 8). In models where Ser 375 was replaced with Trp, His, or Met, the bulky side chains of these residues clashed with NBD-556 in the cavity, whereas the side chain of Phe 43_CD4_, which binds more shallowly than NBD-556 (Figure 2C), did not create a steric clash with Trp 375 (Figure 8B–D). These observations suggest that use of NBD-557 and small molecules inhibitors may exert selection pressure on viruses to replace Ser 375 with Trp, Met, or His as a means to escape neutralization by small molecule inhibitors. However, the rate of Ser 375 to Trp mutation may be counterbalanced because the same mutation is also detrimental to the viruses by predisposing gp120 to adopt the CD4-bound conformation, a state prone to inactivation [21]. Overall, the Phe 43 cavity remains an indispensable target for small molecule inhibitors.

## Discussion

We have determined high-resolution structures of gp120 in complex with small molecules that target the highly conserved CD4-binding site on gp120. Our aim was to ascertain if diverse Region III NBD congeners that exhibit various capacities to enhance viral entry into CD4-negative cells manifest markedly different binding modes. The six structures described herein confirm results from our previous studies [23], [24], [26] that Region I binds deepest within the cavity forming aromatic-aromatic stacking interactions with Trp 427 of gp120 and that shape complementarity between the Region I moiety and cavity can be improved through incorporation of a *meta*-fluorine substituent [21], [27]. These structures also confirm that the two hydrogen bonds formed between the Region II oxalamide linker with backbone carbonyls of Asn 425 and Gly 473 lining the wall of the cavity are an essential determinant of binding affinity for the NBD congeners to gp120. Regarding Region III, which binds to the surface exposed vestibule of the Phe 43 cavity, the NBD-557:gp120 structures indicated that the amine of tetramethylpiperidine ring does not bind Asp 368 and that the contacts with gp120 were not optimal. Indeed, the structures of (*S*)-AS-II-37, (*S*,*S*)-AS-I-261, (*S*,*S*)-MAE-II-167, or (*R*)-MAE-II-188, bound to gp120 exhibited a variety of binding interactions between the Region III moiety and gp120, with only (*S*,*S*)-MAE-II-167 forming a hydrogen bond with Asp 368.

The crystal structures described herein provide insights into further modification of these analogues. The *para*-chloro, *meta*-fluoro aromatic containing congeners improve shape complementarity between Region I of the ligand and the Phe 43 cavity when compared to the initial lead compound NBD-557 (Figure 4A and 4B). The introduction of fluorine in small molecule drugs is a well-known strategy for increasing protein-ligand binding affinity [34], [35]. The structures of the four *meta*-fluoro, *para*-chloro substituted Region I congeners presented here [(*S*)-AS-II-37, (*S*,*S*)-AS-I-261, (*S*,*S*)-MAE-II-167, and (*R*)-MAE-II-188], along with the three previously reported complexes [(*R,R*)- AWS-I-169, (*S,S*)-DMJ-I-228 and (*R,R*)-DMJ-II-121] [24], [26], confirm that interactions between the *meta*-fluoro substituent and Val 255 and Ser 375 in the cavity are associated with increased binding affinity (Figure 4B). In particular, the binding modes of (*S*)-AS-II-37, (*S*,*S*)-AS-I-261, (*S*,*S*)-MAE-II-167 to gp120 suggest that the Region III moiety can be further modified to improve the interactions with Asp 368_gp120_ in Area A (Figure 6). The binding mode of the cyclopropyl-substituted pyrrolidine ring of (*R*)-MAE-II-188, which binds within the opposite face of gp120 vestibule (Area B), provides a strategy to exploit hydrophobic interactions and enhance small molecule-binding affinity (Figure 6). The cyclopropyl ring forms hydrophobic interactions with a groove between the outer-to-inner domain exit loop and the tip of β20/21. As depicted in Figure 6, a compound containing a basic amine that interacts with Asp 368_gp120_ in Area A combined with a hydrophobic moiety that occupies the groove in Area B, may bind with improved affinity. Another area that may be exploited to improve region III-gp120 interactions lies adjacent to Gly 473 (Figure 6, Area C). We previously reported that the incorporation of guanidinium groups into region III (i.e., DMJ-I-228) permitted NBD analogues to achieve mimicry of the Arg 59_CD4_-Asp 368_gp120_ interaction, leading to improved affinities of the small molecules for gp120, along with neutralization potency and breadth against selected clade B and C viruses. Importantly, small molecules that interact with Asp 368_gp120_ did not promote CD4-independent viral entry [24], [26]. Taken together, the six structures of small-molecule complexes with gp120 exhibit diverse binding modes to the Phe 43 cavity and provide novel insights to improve both the affinity and the potency of small molecules against HIV-1 as antiviral agents.

## Materials and Methods

### Purification, crystallization, data collection and refinement

Construction of gp120 core and core_e_ expressing plasmids and purification procedures of the proteins were reported previously [23]. Briefly, the plasmids were transiently transfected to 293f cells and the supernatant containing the proteins secreted were harvested. The supernatant was passed through a 17b-conjugated-Protein A column, washed with PBS, and eluted with Elution buffer (Pierce). The eluent was neutralized to pH 7 with 1 M Tris-HCl, pH 8.0. Then, the proteins were deglycosylated with Endo H, and were further purified with a Concanavalin A column (Sigma) followed by a size exclusion column (Superdex 75 for gp120 core-48d complex and Superdex 200 for gp120 core_e_). Proteins in buffer containing 2.5 mM Tris-HCl pH 7.5, 350 mM NaCl, and 0.02% NaN_3_, were concentrated to ∼15 mg/ml to maintain final concentrations of the small molecules and DMSO for 100 µM and 5%, respectively. The small molecules were synthesized as previously reported [27]. The protein-small molecule complexes were crystallized by the vapor diffusion method, where 0.5 µl of protein solution was mixed with 0.5 µl of the reservoir solution and equilibrated against the reservoir solution containing 10% PEG 8000, 5% iso-propanol and 100 mM HEPES, pH 7.5 for small molecule-clade A/E gp120_93TH057_ H375S core_e_ crystals, and 16–20% PEG 3350 and 100 mM Tris-HCl, pH 7.5 for gp120: NBD-557: 48d complex, respectively. Crystals were soaked in cryo-protectant solution containing 30% Ethylene glycol, 12% PEG 8000, 5% iso-propanol, and 100 mM HEPES, pH 7.5, and were flash frozen in liquid nitrogen prior to data collection. Data were collected at APS ID22, processed, and scaled using HKL2000 [36]. The structure of YU2core-NBD-557-48d complex was solved by molecular replacement using gp120 structure in complex with CD4 and 17b (PDB ID: 1RZK) as a search model, and structures of other small molecule bound-clade A/E gp120_93TH057_ H375S complexes were solved by molecular replacement using AutoMR in PHENIX software suite [37] with unliganded clade A/E gp120 core_e_ (PDB ID: 3TGT) as a search model. Small molecules were manually fitted into the electron density using COOT [38]. The structures were refined with PHENIX and the refinement statistics were summarized in Table 1. Figures were generated with PyMOL [39].

### Surface-plasmon resonance experiments

SPR experiments were performed using Biacore T100 (GE Healthcare) at 25°C. To measure the binding of gp120 core and core_e_ to antibody 48d, 48d fab was immobilized onto a CM5 chip at a density of ∼500 RU using a standard amine coupling chemistry. 50 nM of gp120 core or core_e_ protein was injected over the chip at a flow rate of 40 µl/min and association and disassociation rates were monitored. HBS-EP buffer (GE Healthcare) was used for the running buffer. A capture method was used to monitor the enhancement of gp120 binding to 17b by NBD-analogues. Monoclonal mouse anti-human IgG(Fc) antibody (GE Healthcare) was immobilized onto a CM5 chip at a density of ∼3000 RU, then the antibody 17b IgG was captured on the CM5 chip (∼400 RU). 100 nM of gp120 core or core_e_ with 0–100 µM of each compound was injected over the chip at a flow rate of 40 µl/min. The running buffer contained 20 mM HEPES, pH 7.4, 150 mM NaCl, 0.05% P-20, and 5% DMSO.

### Accession numbers

Coordinates and structure factors for the crystal structures of gp120 in complexes with NBD-analogues have been deposited in the Protein Data Bank. NBD-557: 48d: YU2 gp120 core complex (PDB ID: 4DVR), NBD-557: clade A/E_93TH057_ gp120 core complex (PDB ID: 4DVS), AS-II-37: clade A/E_93TH057_ gp120 core complex (PDB ID: 4DVT), AS-I-261: clade A/E_93TH057_ gp120 core complex (PDB ID: 4DVV), MAE-II-167: clade A/E_93TH057_ gp120 core complex (PDB ID: 4DVW), and MAE-II-188: clade A/E_93TH057_ gp120 core complex (PDB ID: 4DVX).

## Supporting Information

Figure S1 **A modified YU2 gp120 core (core V3s) makes a stable interaction with antibody 48d.** (A) Profiles of two Superdex 75 column runs on FPLC. Blue cure represents an elution profile of YU2core:48d:NBD-557 complex in the presence of 50 µM NBD-557 in the running buffer (2.5 mM Tris-HCl pH 7.5, 350 mM NaCl, 0.02% NaN3, 5% DMSO, and 50 µM NBD-557). The red curve represents the profile of YU2 gp120 core (V3s):48d:NBD-557. The front peak of each profile contained the complex. The second peak contained unbound Fab 48d. (B) SPR sensograms showing association and disassociation of 50 nM YU2 core and YU2 core (V3s) to the ligand, 48d fab, which was directly immobilized onto a CM5 chip.
(TIF)

Figure S2 **Three possible Region III conformations of AS-II-37 in** ***2Fo-Fc*** **electron density map.** The conformation C was chosen for analysis, where the nitrogen atom in the Region III is positioned to make a hydrogen bond with Asp 368_gp120_.
(TIF)

Figure S3 **The preferred gp120-binding enantiomers determined by crystallography.** The gp120-bound crystal structures obtained from racemic mixtures of the NBD-analogues revealed preferential binding of (*S*)-AS-II-37, (*S,S*)-AS-I-261, (*S,S*)-MAE-II-167, and (*R*)-MAE-II-188.
(TIF)

Table S1 **Biological activity of NBD analogues.** IC_50_s, activation of viral infectivity, and binding affinities for NBD analogues are summarized. Data for **1**, **2** and **3** were previously reported by Madani et al. [21].
(DOCX)
