# Supplementary figures and images for: Crystal Structures of HIV-1 gp120 Envelope Glycoprotein in Complex with NBD Analogues That Target the CD4-Binding Site
Source: PLoS One. 2014 Jan 28;9(1):e85940. doi: 10.1371/journal.pone.0085940 (PMC3904841; doi:10.1371/journal.pone.0085940)

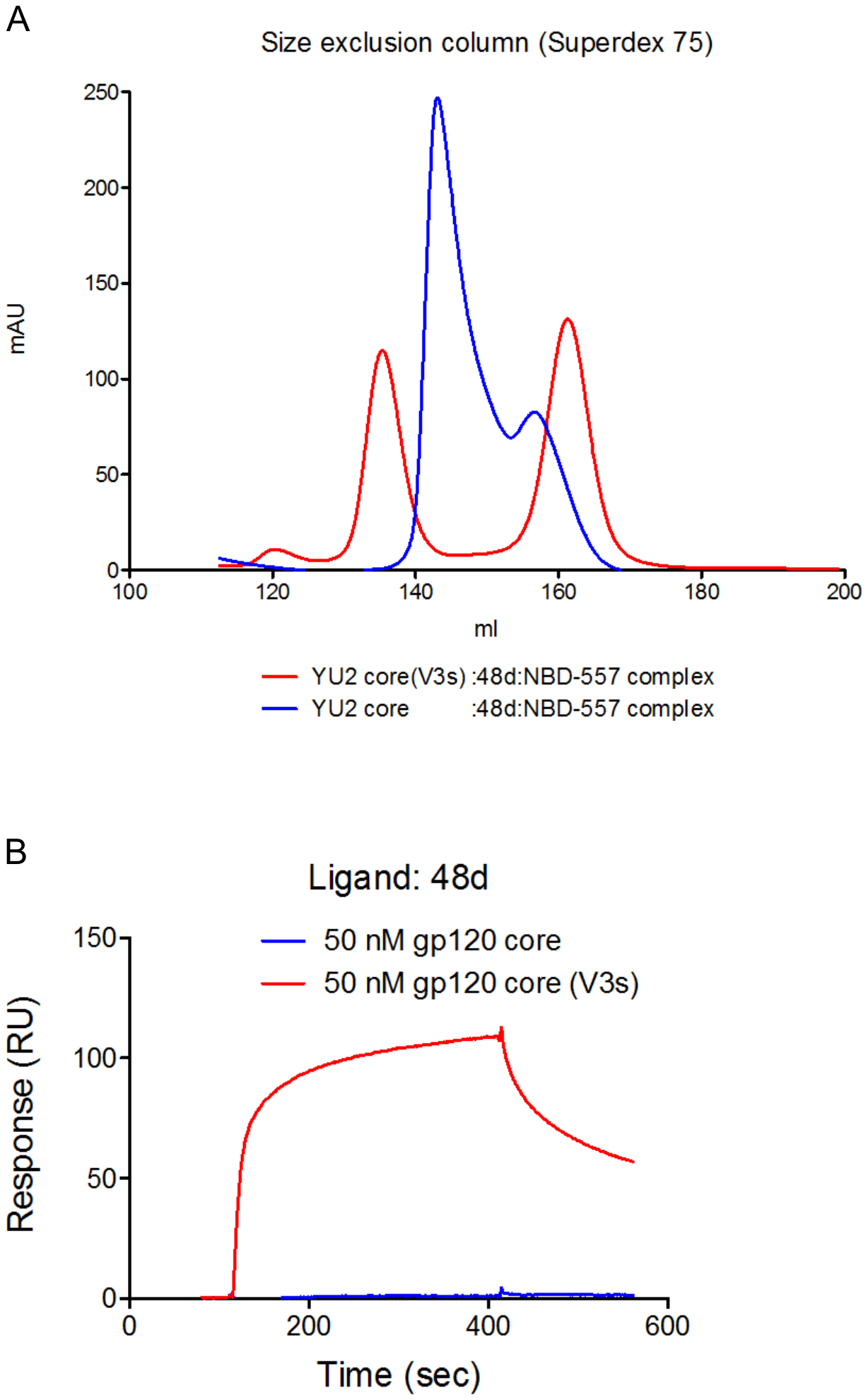

Supplement: Figure S1 — A modified YU2 gp120 core (core V3s) makes a stable interaction with antibody 48d. (A) Profiles of two Superdex 75 column runs on FPLC. Blue cure represents an elution profile of YU2core:48d:NBD-557 complex in the presence of 50 µM NBD-557 in the running buffer (2.5 mM Tris-HCl pH 7.5, 350 mM NaCl, 0.02% NaN3, 5% DMSO, and 50 µM NBD-557). The red curve represents the profile of YU2 gp120 core (V3s):48d:NBD-557. The front peak of each profile contained the complex. The second peak contained unbound Fab 48d. (B) SPR sensograms showing association and disassociation of 50 nM YU2 core and YU2 core (V3s) to the ligand, 48d fab, which was directly immobilized onto a CM5 chip. (TIF) [file pone.0085940.s001.tif]

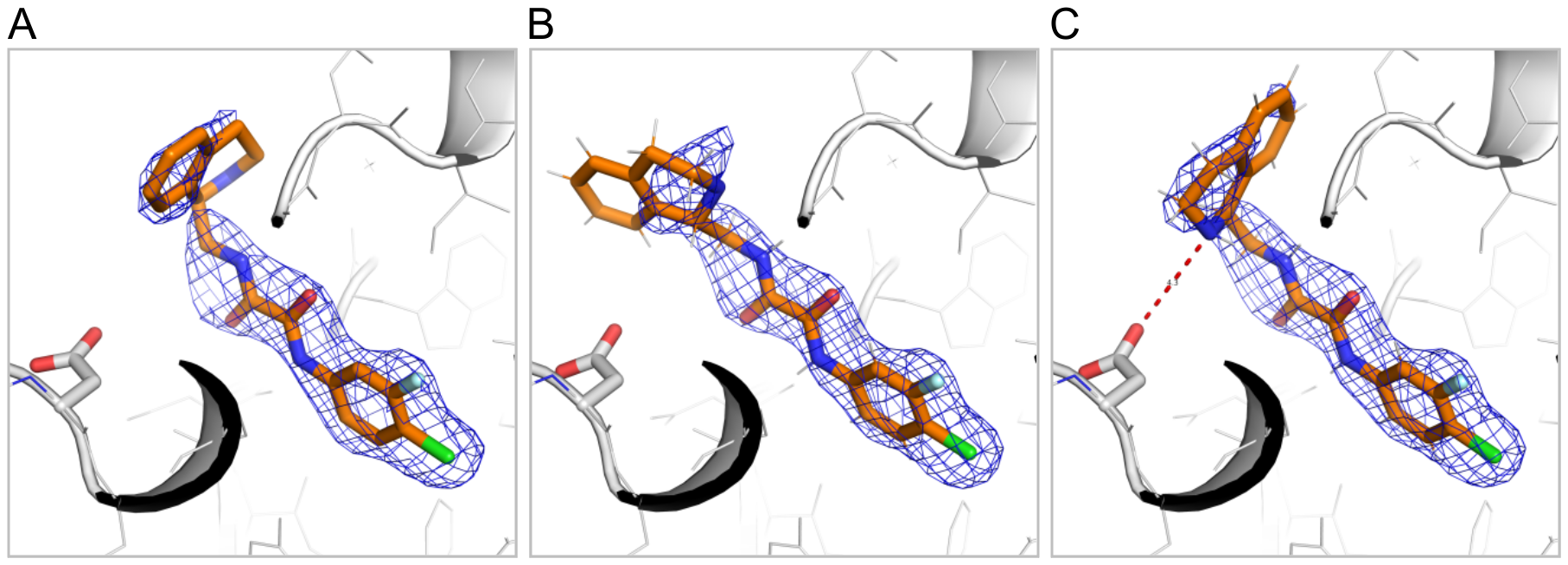

Supplement: Figure S2 — Three possible Region III conformations of AS-II-37 in 2Fo-Fc electron density map. The conformation C was chosen for analysis, where the nitrogen atom in the Region III is positioned to make a hydrogen bond with Asp 368gp120. (TIF) [file pone.0085940.s002.tif]

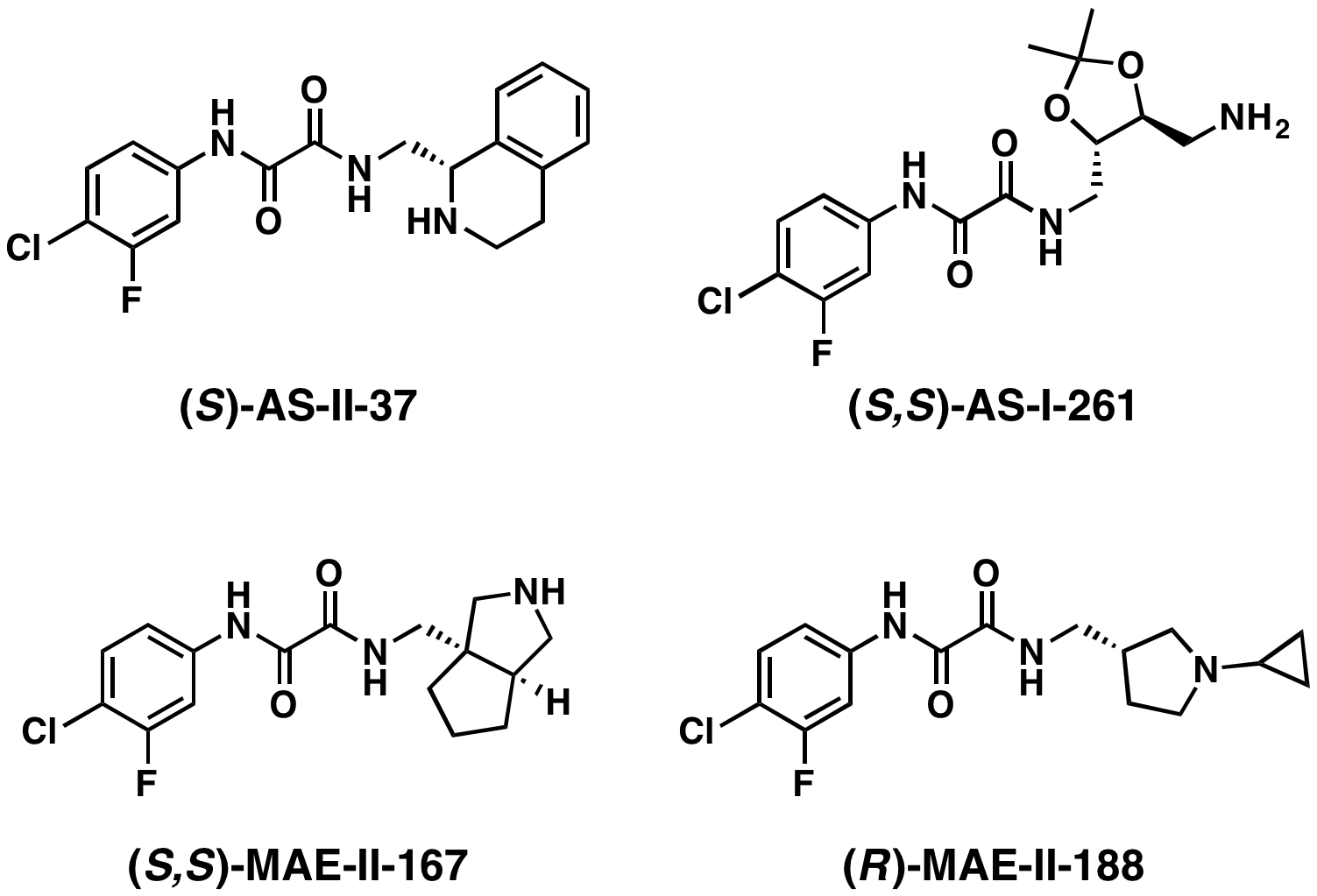

Supplement: Figure S3 — The preferred gp120-binding enantiomers determined by crystallography. The gp120-bound crystal structures obtained from racemic mixtures of the NBD-analogues revealed preferential binding of (S)-AS-II-37, (S,S)-AS-I-261, (S,S)-MAE-II-167, and (R)-MAE-II-188. (TIF) [file pone.0085940.s003.tif]
