# Supplementary material for: Crystal Structures of HIV-1 gp120 Envelope Glycoprotein in Complex with NBD Analogues That Target the CD4-Binding Site
Source: PLoS One. 2014 Jan 28;9(1):e85940. doi: 10.1371/journal.pone.0085940 (PMC3904841; doi:10.1371/journal.pone.0085940)
Supplement: Table S1 — Biological activity of NBD analogues. IC50s, activation of viral infectivity, and binding affinities for NBD analogues are summarized. Data for 1, 2 and 3 were previously reported by Madani et al. [21]. (DOCX) [file pone.0085940.s004.docx]

**Table S1. Biological activity of NBD analogues.**

| Compound | HIV-1, YU2  IC_50_ (μM)^a^ | A-MLV  IC_50_ (µM)^b^ | Activation of  Viral Infectivity^c^ | *K*_D_  (μM)^d^ |
| --- | --- | --- | --- | --- |
| 1 NBD-556 | >100 | >100 | 1.0 + 0.1 | 3.70 |
| 2 NBD-557 | 3.08 + 0.6* | N.D. | 0.9 + 0.4 | 2.20 |
| 3 JRC-II-191 | 54.4 | >100 | 1.9 + 0.2 | 0.76 |
| 4 AS-II-137 | 50.4 +14.2 | >100 | 0.6 | 1.2 |
| 5 AS-I-261 | 76.6 + 5.7 | 94.4 | 1.5 | 2.6 |
| 6 MAE-II-167 | 68.3 + 9.6 | 93.1 | 0.0 | 3.1 |
| 7 MAE-II-188 | 33.5 + 9.1 | >100 | 0.3 | 1.7 |
| 8 AWS-I-169 | 21.3 + 5.0 | >100 | 0.0 | 0.30 |
| 9 DMJ-I-228 | 22.9 + 2.4 | >100 | 0.0 | 0.25 |
| 10 DMJ-II-121 | 2.3 + 0.05 | >100 | 0.0 | 0.11 |

^a-d^ Data for **1**, **2** and **3** as previously reported by Madani *et al.*[21]. *The IC_50_ for **2** as reported by Zhao *et al*. [18] was determined in the laboratory-adapted HIV-1_IIIB_ strain. Data for **4**-**10** were previously published [24,27]. Briefly, ^a^the IC_50_ was determined in Cf2Th-CD4/CCR5 cells infected with HIV-1 YU2 virus. ^b^The IC_50_ in cells infected with amphotropic murine leukemia virus (A-MLV). ^c^The relative activation of viral infectivity in CD4 negative Cf2Th-CCR5 cells infected with HIV-1YU2 virus normalized to **1**  for **2**, **3** and **10**; the relative infectivity **4-7** were normalized to **2** and the relative infectivity **8** and **9** were normalized to N-(4-Chloro-3-Fluorophenyl)-N'-(1,2,2,6,6-Pentamethylpiperidin-4-yl)ethanediamide as described [25]. N.D. not determined.
